# Garcinol Sensitizes NSCLC Cells to Standard Therapies by Regulating EMT-Modulating miRNAs

**DOI:** 10.3390/ijms20040800

**Published:** 2019-02-13

**Authors:** Mohd Farhan, Arshi Malik, Mohammad Fahad Ullah, Sarah Afaq, Mohd Faisal, Ammad Ahmad Farooqi, Bernhard Biersack, Rainer Schobert, Aamir Ahmad

**Affiliations:** 1 College of Basic Sciences, King Faisal University, Hofuf, 400, Al Ahsa 31982, Saudi Arabia; mfarhan@kfu.edu.sa; 2 Clinical Biochemistry, College of Medicine, King Khalid University, Abha 62529, Saudi Arabia; amalik@kku.edu.sa (A.M.); safaq@kku.edu.sa (S.A.); 3 Department of Medical Laboratory Technology, Faculty of Applied Medical Science, University of Tabuk, Tabuk 71491, Saudi Arabia; m.ullah@ut.edu.sa; 4 Department of Psychiatry, University Hospital Limerick, Limerick V94 T9PX, Ireland; mohd.faisal@hse.ie; 5 Institute of Biomedical and Genetic Engineering (IBGE), Islamabad 44000, Pakistan; ammadfarooqi@rlmclahore.com; 6 Organic Chemistry Laboratory, Universitätsstr. 30, 95447 Bayreuth, Germany; bernhard.biersack@yahoo.com (B.B.); rainer.schobert@uni-bayreuth.de (R.S.); 7 Department of Pathology, Wayne State University and Karmanos Cancer Institute, Detroit, MI 48201, USA

**Keywords:** garcinol, NSCLC, EMT, erlotinib, cisplatin, miRNAs

## Abstract

Garcinol, a dietary factor obtained from *Garcinia indica*, modulates several key cellular signaling pathways as well as the expression of miRNAs. Acquired resistance to standard therapies, such as erlotinib and cisplatin, is a hallmark of non-small cell lung cancer (NSCLC) cells that often involves miRNA-regulated epithelial-to-mesenchymal transition (EMT). We used A549 cells that were exposed to transforming growth factor beta 1 (TGF-β1), resulting in A549M cells with mesenchymal and drug resistant phenotype, and report that garcinol sensitized resistant cells with mesenchymal phenotype to erlotinib as well as cisplatin with significant decrease in their IC_50_ values. It also potentiated the apoptosis-inducing activity of erlotinib in A549M and the endogenously mesenchymal H1299 NSCLC cells. Further, garcinol significantly upregulated several key EMT-regulating miRNAs, such as miR-200b, miR-205, miR-218, and let-7c. Antagonizing miRNAs, through anti-miRNA transfections, attenuated the EMT-modulating activity of garcinol, as determined by mRNA expression of EMT markers, E-cadherin, vimentin, and Zinc Finger E-Box Binding Homeobox 1 (ZEB1). This further led to repression of erlotinib as well as cisplatin sensitization, thus establishing the mechanistic role of miRNAs, particularly miR-200c and let-7c, in garcinol-mediated reversal of EMT and the resulting sensitization of NSCLC cells to standard therapies.

## 1. Introduction

**Figure 1:**
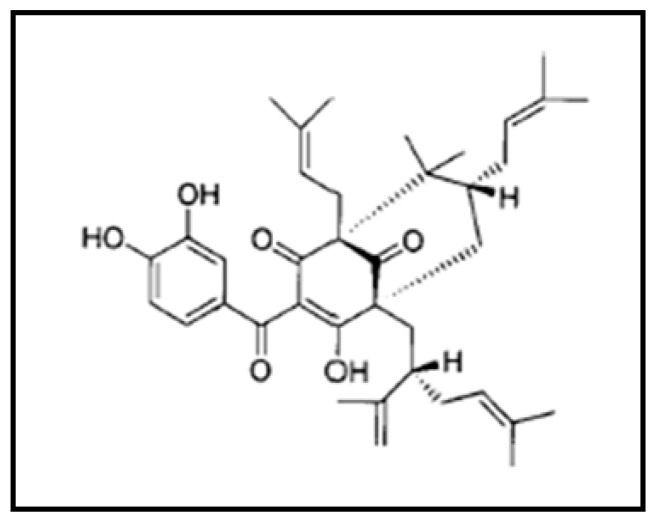
Structure of garcinol.

Garcinol (Figure 1) is isolated from the “Kokum” plant (*Garcinia indica*) that grows extensively on the western coast of India [1]. The genus *Garcinia* includes some 200 species found in the tropics, especially Asia and Africa. *Garcinia indica* extracts, particularly those from its rind, are rich in polyisoprenylated benzophenone derivatives, including garcinol. In our earlier studies on the anticancer property of garcinol, we demonstrated modulation of NF-κB (nuclear factor kappa-light-chain-enhancer of activated B cells) signaling by garcinol, leading to induction of apoptosis in receptor positive and triple negative breast cancer cells [2], as well as prostate and pancreatic cancer cells [3]. In recent years, there has been a lot of interest in characterizing the anticancer role of garcinol in different human cancers [4,5,6,7,8].

Among all the cancers that affect humans, lung cancer is the leading cause of cancer-related deaths. In the United States, it accounts for a quarter of all cancer deaths and is expected to result in approximately 154,050 deaths this year [9]. Globally, lung cancer results in about 1.2 million deaths a year [10]. With these staggering numbers, there has been interest in evaluating the anticancer effects of garcinol in lung cancer models as well. First, it was shown that garcinol can induce cell cycle arrest in H1299 non-small cell lung cancer (NSCLC) cells [11]. More recently, garcinol has been shown to suppress stemness in NSCLC A549 cells through its action on Wnt/β-catenin/ Signal transducer and activator of transcription 3 (STAT3) signaling [12] and Aldehyde Dehydrogenase 1 Family Member A1 (ALDH1A1) expression [13]. Incidentally, H1299 cells represent mesenchymal phenotype and we have earlier reported a role of hedgehog signaling in maintenance of mesenchymal phenotype and the stemness of NSCLCs with the targeting of hedgehog signaling resulting in sensitization of NSCLCs to standard chemotherapies [14].

Epithelial-to-mesenchymal transition (EMT), regulated by various signaling pathways as well as microRNAs (miRNAs) [15], is an attractive target for lung cancer therapy and the reversal of therapy resistance [16]. Although we have reported regulation of miRNAs by garcinol in breast cancer cells with resulting regulation of EMT [17], such regulation of miRNAs and/or EMT by garcinol in lung cancer models has never been investigated. In particular, it has never been tested if garcinol can reverse EMT in NSCLC cells thereby resulting in re-sensitization of otherwise resistant cells. To fill this void in our understanding, we tested the anti-proliferation and apoptosis-inducing effects of garcinol on mesenchymal H1299 as well as the A549M cells, the mesenchymal variants of parental A549 NSCLC cells that are rendered mesenchymal by exposure to transforming growth factor beta 1 (TGF-β1) with resulting resistance against standard therapies such as tyrosine kinase inhibitor (TKI) erlotinib and cisplatin. Further, we also investigated the mechanistic role of select miRNAs in the EMT regulation of therapy resistance, as well as their modulation by garcinol.

## 2. Results

### 2.1. Garcinol Sensitizes Resistant Cells to Erlotinib and Cisplatin

**Figure 2:**
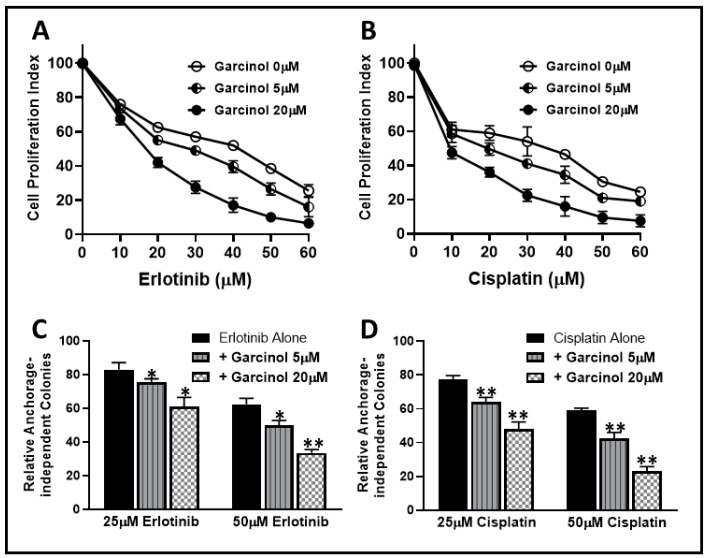
Garcinol sensitizes transforming growth factor beta 1 (TGF-β1)-induced epithelial-to-mesenchymal transition (EMT) cells, A549M to therapy. A549M cells were treated with increasing doses of erlotinib (**A**) and cisplatin (**B**) in the absence (garcinol 0 μM) as well as presence of increasing doses of garcinol (5 and 20 μM) for 72 h and then subjected to 3-(4,5-dimethylthiazol-2-yl)-2,5-diphenyltetrazolium bromide (MTT) assay. (**C**,**D**) The effect of such treatment on anchorage-independent colony formation was also observed. The number of colonies are represented as %, relative to the control conditions with no erlotinib/cisplatin or garcinol. * *p* < 0.05 and ** *p* < 0.01, compared to erlotinib alone.

In our earlier work [14], we established that NSCLC A549 cells undergo EMT when exposed to TGF-β1. The mesenchymal phenotypic A549M cells were also markedly resistant to standard chemotherapies such as erlotinib and cisplatin. As reported in that study, the erlotinib as well as cisplatin IC_50_ and IC_90_ values for A549M cells were significantly higher, relative to the parental A549 cells. IC_50_ values increased from 11.6 to 43.6 μM for erlotinib and from 4.1 to 36.2 μM for cisplatin. In view of these observations, we used A549M cells as our model of chemo-resistant cells and tested the ability of garcinol to possibly sensitize A549M cells to erlotinib and cisplatin. We first treated A549M cells with increasing doses of erlotinib for 72 h in the absence and presence of two different doses of garcinol (5 and 20 μM). As seen in Figure 2A, garcinol at both the tested doses resulted in sensitization to erlotinib treatment. We also calculated the drop in IC_50_ values and found that 5 μM garcinol treatment resulted in 32.95% decrease in IC_50_ value while the higher dose of 20 μM resulted in a decrease in IC_50_ value by 60.37% (Table 1).

Having observed a significant effect of garcinol on sensitization of A549M cells towards erlotinib, we next tested the effect of garcinol treatment on cisplatin sensitivity. This time, we exposed A549M cells to increasing doses of cisplatin for 72 h in the absence and presence of the same two doses of garcinol (5 and 20 μM). We observed that garcinol at both doses sensitized A549M cells to cisplatin (Figure 2B). The IC_50_ of cisplatin in A549M cells was calculated to be 36.5 μM but it dropped to 19.2 μM in the presence of 5 μM garcinol (47.40% decline) and further to 9.1 μM in the presence of 20 μM garcinol (75.07% decline) (Table 1).

Next, we tested the sensitizing effect of garcinol on the clonogenicity of A549M cells when treated with erlotinib or cisplatin, as assayed by the soft agar assay that evaluates anchorage-independent clonogenicity. We observed that, similar to proliferation assays above, garcinol significantly potentiated the inhibitory effect of erlotinib (Figure 2C) as well as cisplatin (Figure 2D) on clonogenic potential of A549M cells. Combined, these results established that garcinol had a very significant effect on the sensitization of A549M cells to erlotinib as well as cisplatin.

### 2.2. Garcinol Potentiates Apoptosis Induction by Erlotinib

**Figure 3:**
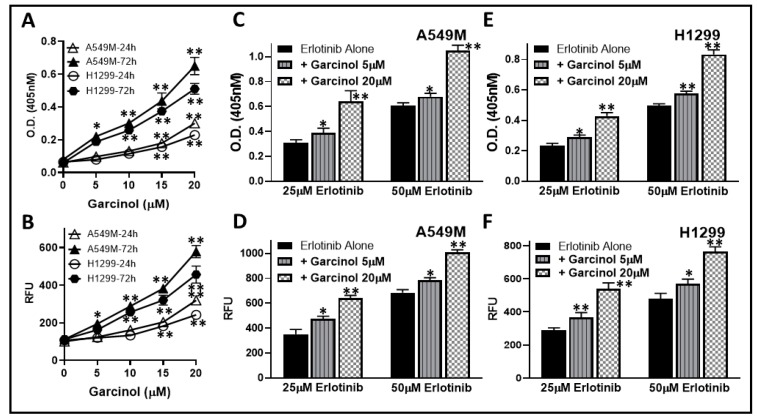
Garcinol potentiates apoptosis induction. A549M and H1299 cells were treated with increasing doses of garcinol for 24 h/72 h, and the induction of apoptosis was measured by Histone/DNA enzyme-linked immunosorbent assay (ELISA) (**A**) and homogeneous caspase-3/7 assay (**B**). * *p* < 0.05 and ** *p* < 0.01, compared to 0 μM garcinol. A549M (**C**,**D**) and H1299 (**E**,**F**) cells were treated with 25 and 50 μM erlotinib for 24 h in the absence (erlotinib alone) or presence of 5 and 20 μM garcinol, and the apoptosis was detected by Histone/DNA ELISA (**C**,**E**) and homogenous caspase-3/7 assay (**D**,**F**). * *p* < 0.05 and ** *p* < 0.01, compared to erlotinib alone.

Targeted chemotherapy, erlotinib, induces apoptosis in NSCLC cells. Since we observed that garcinol sensitized resistant A549M cells, the cells with induced EMT, to erlotinib treatment, we next questioned if this was due to some effect of garcinol on apoptosis inducing ability of erlotinib. We first tested the effect of garcinol alone on apoptosis induction in A549M cells as well as the H1299 cells that have mesenchymal phenotype. Induction of apoptosis was assessed by two different methods—the histone/DNA enzyme-linked immunosorbent assay (ELISA) and caspase-3/7 assay. First, we tested the ability to garcinol to induce apoptosis in these two cell lines. As seen in Figure 3A,B, we observed a dose-dependent induction of apoptosis by garcinol, as assessed by both the methodologies. We evaluated the apoptosis-inducing activity using two different time points—24 h and 72 h—and a clear time-dependent increase in apoptosis-induction in both the cell lines was observed (Figure 3A,B). Next, we selected two different doses of erlotinib (25 and 50 μM) and used these doses on the two cell lines in the absence and presence of two different doses of garcinol (5 and 20 μM). In A549M cells (Figure 3C,D), we observed apoptosis induction by both the doses of erlotinib with more significant induction of apoptosis by the higher (50 μM) dose. Further, the addition of garcinol at both doses potentiated the apoptosis induction with the potentiation much more significant (*p* < 0.001) at the higher dose of garcinol (20 μM). We performed similar experiments in H1299 cells (Figure 3E,F) and confirmed the potentiation of apoptosis-inducing activity of erlotinib by garcinol in these cells as well. The combination of garcinol and erlotinib was observed to induce approximately similar apoptosis within 24 h as was induced by garcinol alone in 72 h. These results in two different cell lines, using two different assays, confirmed that the sensitization of resistant NSCLC cells to chemotherapy involve potentiation of apoptosis induction by garcinol.

### 2.3. Garcinol Affects the Expression of EMT-Modulating miRNAs

**Figure 4:**
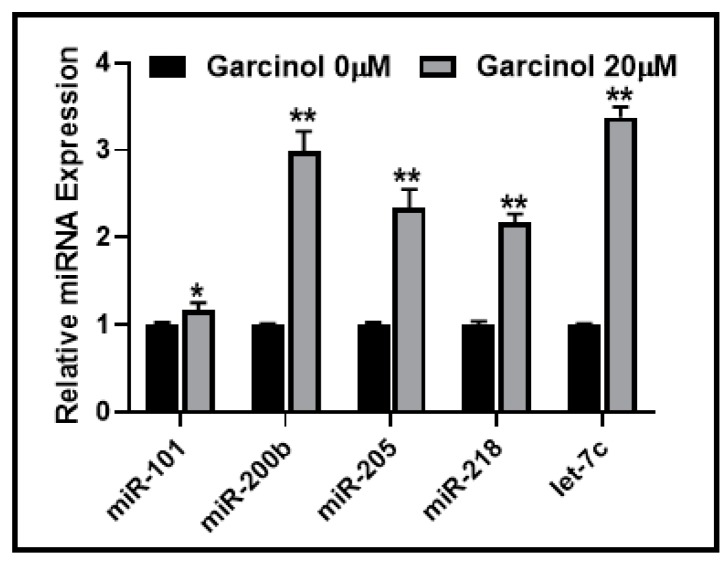
Garcinol upregulates EMT-regulating miRNAs. A549M cells were treated with 20 μM garcinol for 72 h and the expression of select miRNAs was evaluated relative to control cells (garcinol 0 μM). * *p* < 0.05, ** *p* < 0.01.

To further explore the mechanism by which garcinol may sensitize resistant NSCLC cells with mesenchymal phenotype, we focused on miRNAs as these small non-coding RNAs are now well-established regulators of EMT. We shortlisted a few miRNAs for our mechanistic analyses. miR-200b and let-7c were chosen for their role in induction of EMT as reported by us earlier [14]. miR-218 and miR-101 were chosen for their roles in EMT induction and resulting cisplatin resistance in lung cancer cells [18,19], while miR-205 was shortlisted because of the report on its role in EMT regulation and erlotinib resistance in NSCLC cells [20]. Our goal was to study the effect, if any, on the expression of these miRNAs by garcinol. We hypothesized that regulation of these miRNAs by garcinol can profoundly affect EMT and thus be responsible for the observed sensitizing effects of resistant NSCLC cells. When treated with 20 μM garcinol, we observed a significant upregulation of all of these miRNAs in A549M cells (Figure 4). While significant (*p* < 0.05), the upregulation of miR-101 was found to be the least among all the tested miRNAs. All the other miRNAs (miR-200b, miR-205, miR-218, and let-7c) were much more upregulated (*p* < 0.01) with miR-200b and let-7c being the most significantly upregulated miRNAs.

### 2.4. Effects of Garcinol on EMT Are Attenuated by Anti-miRNAs

**Figure 5:**
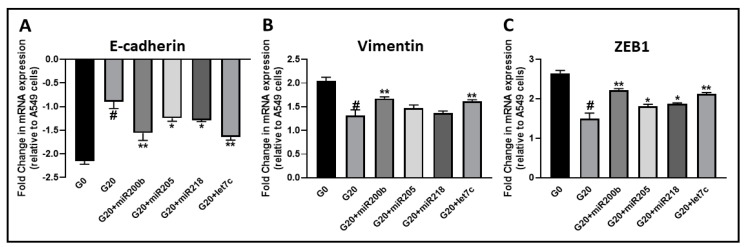
Expression of EMT markers. Expression of epithelial marker E-cadherin (**A**) and mesenchymal markers vimentin (**B**) and ZEB1 (Zinc Finger E-Box Binding Homeobox 1) (**C**) was measured in A549M cells, relative to parental A549 cells. G0: control untreated cells, G20: cells treated with 20 μM garcinol for 72 h, G20+miR-200b: cells treated with 20 μM garcinol for 72 h after transfections with anti-miR-200b, G20+miR-205: cells treated with 20 μM garcinol for 72 h after transfections with anti-miR-205, G20+miR-218: cells treated with 20 μM garcinol for 72 h after transfections with anti-miR-218, G20+let7c: cells treated with 20 μM garcinol for 72 h after transfections with anti-Let-7c. # *p* < 0.01, compared to G0, * *p* < 0.05, relative to G20, ** *p* < 0.01, relative to G20.

We further established the mechanistic involvement of miRNAs in EMT regulation by garcinol through transfections with anti-miRNA oligos. A549M cells were either transfected with non-specific or specific anti-miRNAs, and treated with 20 μM garcinol for 72 h. As seen in Figure 5A, control A549M cells had considerably reduced E-cadherin (a marker of epithelial phenotype) expression, as compared to parental A549 cells. Treatment with garcinol significantly attenuated this E-cadherin downregulation, probably because of the increased expression of miRNAs as reported above. To confirm the role of miRNAs, we transfected A549M cells with anti-miRNAs before garcinol treatment and observed that antagonizing all miRNAs had significant effect on the garcinol activity, albeit to different extents. We further confirmed these results by assessing the markers of mesenchymal phenotype namely vimentin (Figure 5B) and ZEB1 (Figure 5C). In contrast to E-cadherin, the expression of vimentin and ZEB1 was increased in EMT-induced A549M cells, as expected. Garcinol treatment resulted in a significant decrease in the expression of these mesenchymal markers, thus directly verifying an EMT-reversing effect. Further, anti-miRNA transfections significantly attenuated the garcinol effects, further attesting to the functional role of these miRNAs in EMT-reversing activity of garcinol. Of note, miR-200b and let-7c were found to be the most effective miRNA in terms of attenuating garcinol activity.

### 2.5. Erlotinib and Cisplatin Sensitizing Activity of Garcinol is Affected by Anti-miRNAs

**Figure 6:**
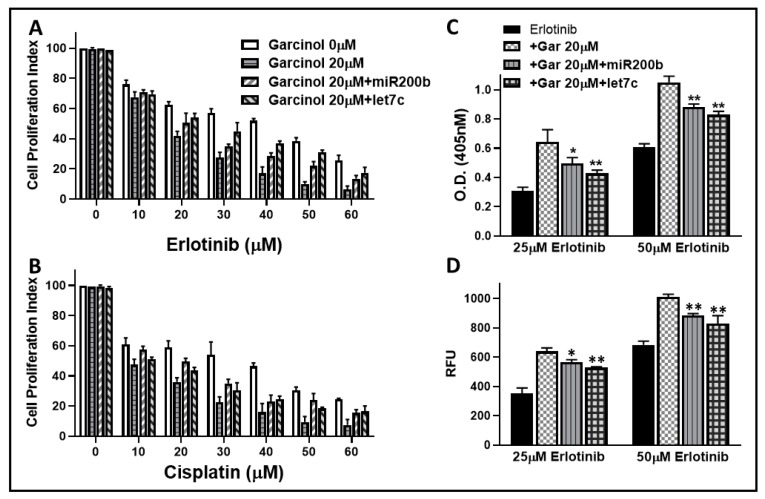
Mechanistic role of miRNAs in drug sensitization. Effect of anti-miRNAs was evaluated on garcinol-induced sensitization of A549M cells to erlotinib (**A**) and cisplatin (**B**), through MTT assay. Treatment with drugs in the absence (garcinol 0 μM) or presence of 20 μM garcinol was for 72 h, after transfections with non-specific or specific anti-miRNAs. Effect of anti-miRNAs was also evaluated on potentiation of apoptosis induction as assayed by Histone/DNA ELISA (**C**) and homogeneous caspase-3/7 assay (**D**). * *p* < 0.05 and ** *p* < 0.01, compared to garcinol 20 μM.

Since we observed that miRNAs, particularly miR-200b and let-7c, significantly attenuated the effects of garcinol on EMT markers, we next tested whether this regulation of EMT through miRNAs could also define the drug-sensitizing activity of garcinol. Therefore, we again exposed A549M cells to increasing doses of either erlotinib (Figure 6A) or cisplatin (Figure 6B) in the presence of garcinol, and further added the anti-miR-200b or the anti-let-7c in the individual experiments. While 20 μM garcinol sensitized the cells to erlotinib as well as cisplatin at all of their tested doses, addition of anti-miRNA significantly altered such sensitizing ability of garcinol. Interestingly, let-7c was most effective at attenuating the effects on erlotinib sensitivity while both miR-200b and let-7c were equally effective at attenuating the effects on cisplatin sensitivity with mR-200b perhaps slightly more effective.

Further, in addition to assessing proliferation of cells as a surrogate for drug activity, we also tested the effects of anti-miRNAs on the garcinol-induced potentiation of erlotinib-induced apoptosis in A549M cells for the further verification of our results. Through DNA/histone ELISA assay (Figure 6C) as well as homogenous caspase-3/7 assay (Figure 6D), we confirmed that whereas 20 μM garcinol potentiated the apoptosis inducing activity of erlotinib (at the both the tested doses of erlotinib), addition of anti-miRNAs significantly attenuated the garcinol activity. 

## 3. Discussion

The major findings from our study are: (i) garcinol sensitizes drug resistant NSCLC cells with EMT phenotype to cytotoxic effects of erlotinib as well as cisplatin; (ii) such sensitizing activity of garcinol involves potentiation of apoptosis-inducing activity of chemotherapy; (iii) garcinol induces the expression of tumor-suppressive miRNAs that regulate EMT; and (iv) the miRNAs, particularly miR-200b and let-7c, are mechanistically involved in EMT suppressing and drug sensitizing activity of garcinol.

Resistance to chemotherapy is a major clinical challenge. Erlotinib, marketed as “Tarceva” is a first-generation EGFR-TKI approved by the US Food and Drug Administration (FDA) for treatment of locally advanced or metastatic NSCLC. Cisplatin belongs to the family of platinum-based drugs that bind to DNA and inhibits replication. While both of these drugs are used in clinics for the treatment of lung cancer patients, their continued use often results in acquired resistance [21,22]. While a number of factors are now being evaluated for possible roles in acquired chemoresistance, the phenomenon of EMT remains one of the leading underlying mechanism. This was confirmed by us when we observed a role of hedgehog signaling in EMT induction and resulting chemoresistance in NSCLC cells [14]. Taking a cue from this earlier published study, we now assessed the role of garcinol, a naturally occurring chemopreventive agent, to reverse EMT and the resulting chemoresistance. We hypothesized that garcinol might be an effective agent for such assessment, based on our results in a study focused on breast cancer cells where we observed EMT reversing effects of garcinol [17].

Our results are the first to establish a sensitizing role of garcinol in drug resistant NSCLC models. Previously, garcinol has been shown to alter miRNA signature and sensitize human pancreatic cancer cells to gemcitabine [23] as well as sensitize breast cancer cells to taxol in vivo in a mouse breast cancer cell model [24]. Interestingly, both of these studies hinted at the possible modulation of NF-κB signaling, which was earlier shown by us to be modulated by garcinol in breast, prostate, and pancreatic cancer cells [2,3]. NF-κB signaling is intricately connected with cancer drug resistance [25], and therefore its modulation by garcinol might be of importance in the context of drug resistance. Another major signaling involved in EMT and cancer drug resistance is the STAT3 signaling [26], and our own results [27], followed by reports from other research groups [7,12,28,29], established potent activity of garcinol against STAT3 activation, which might be another mechanism through which garcinol can effectively regulated EMT and the resistance to chemotherapy.

In addition to cellular signaling pathways, miRNAs are increasingly being sought as the regulators of EMT and drug resistance. Some of the better characterized miRNAs that regulate EMT are the tumor suppressors ones such as the miR-200 family, let-7c family, miR-205, miR-101, miR-218, etc. These miRNAs facilitate maintenance of epithelial and less aggressive phenotypes, and are therefore, often found downregulated in drug resistance and metastatic cancers. We observed an upregulation of these miRNAs as a result of treatment with garcinol, which might explain the EMT reversing activity of garcinol and the resulting sensitization to erlotinib as well as cisplatin. To further tie these miRNAs mechanistically with the sensitizing activity of garcinol, we used anti-miRNAs with the hypothesis that antagonizing miRNAs will attenuate the activity of garcinol. Indeed, we observed that blocking the upregulation of these tumor suppressive and EMT-inhibiting miRNAs by garcinol, reverses the effects of garcinol on EMT as well as the sensitization to erlotinib and cisplatin.

Use of natural agents, such as garcinol, in cancer therapy has been advocated primarily based on the promising pre-clinical results and the knowledge that such dietary agents are well tolerated with minimal to no toxic effects along with their pleiotropic effects [30]. However, a major challenge in the clinical advancement of such agents is poor bioavailability. One strategy to overcome this is through synthesis of synthetic garcinol analogs with enhanced bioavailability as well as efficacy [31]. Also, nanoformulations of garcinol have been found to be a promising strategy to improve its bioavailability [32]. The efficacy of garcinol as a promising anticancer agent has been demonstrated in several investigations that used in vivo models representing various human cancers [12,17,24,27,29,32,33,34].

In conclusion, the ability of garcinol to regulate miRNAs and EMT with resulting effects on sensitization of resistant NSCLC cells is a novel finding that we report here with the hope that future investigations will further evaluate the anticancer potential of garcinol in NSCLC as well as other relevant cancers in pre-clinical and clinical studies.

## 4. Materials and Methods

### 4.1. Cell Lines and Reagents

The human lung adenocarcinoma cell lines A549 and H1299 were purchased from the American Type Culture Collection (Manassas, VA, USA) and maintained according to the American Type Culture Collection’s instructions. All cells were cultured in 5% CO_2_–humidified atmosphere at 37 °C. A549 cells were treated with TGF-β1 (5 ng/mL) for 3 weeks to generate A549M cells (EMT phenotype cells). Garcinol was isolated and purified using published methods [35] and the results validated by using commercially available garcinol from Biomol/Enzo Life Sciences International, Inc. (Plymouth Meeting, PA, USA).

### 4.2. Cell Proliferation Studies

Cells were seeded at a density of 2 × 10^3^ cells per well overnight. Thereafter, culture media was removed and replaced with a fresh media containing DMSO (dimethyl sulfoxide: vehicle control) or different concentrations of garcinol diluted from a 25 mM stock. After an incubation of 72 h, 25 μL of 3-(4,5-dimethylthiazol-2-yl)-2,5-diphenyltetrazolium bromide (MTT) solution (5 mg/mL in phosphate-buffered saline, PBS) was added to each well and incubated further for 2 h at 37 °C. Finally, supernatant was aspirated and the MTT formazan, formed by metabolically viable cells, was dissolved in DMSO (100 μL) by gentle mixing for 30 min on a gyratory shaker. The absorbance was measured at 595 nm on an Ultra Multifunctional Microplate Reader (TECAN, Durham, NC, USA). Each treatment had eight replicate wells and the amount of DMSO in reaction mixture never exceeded 0.1%. Moreover, each experiment was repeated at least three times.

### 4.3. Soft Agar Colonization Assay

A549M cells (3 × 10^4^) were first treated with erlotinib alone or erlotinib in the presence of increasing concentrations of garcinol for 72 h, and then collected by trypsinization. Cells were counted and 3 × 10^4^ cells were plated in 0.5 mL of culture medium containing 0.3% (*w*/*v*) top agar layered over a basal layer of 0.7% (*w*/*v*) agar (with culture medium and the supplements) in 24-well plates. Culture medium was supplemented with erlotinib alone or erlotinib and different concentrations of garcinol even during this seeding in soft agar. Plates were incubated for 2 weeks and colonies were counted. All assays were carried out in quadruplicate, and results are representative of three independent observations.

### 4.4. Histone/DNA ELISA for Apoptosis

The Cell Death Detection ELISA Kit (Roche, Palo Alto, CA, USA) was used to detect apoptosis, as described previously [3]. Briefly, after appropriate treatment, cytoplasmic histone/DNA fragments from cells were extracted and incubated in the microtiter plate modules coated with antihistone antibody. Subsequently, the peroxidase-conjugated anti-DNA antibody was used for the detection of immobilized histone/DNA fragments followed by color development with ABTS (2,2′-azino-bis(3-ethylbenzothiazoline-6-sulphonic acid)) substrate for peroxidase. The spectrophotometric absorbance of the samples was determined by using Ultra Multifunctional Microplate Reader (TECAN) at 405 nm.

### 4.5. Homogeneous Caspase-3/7 Assay for Apoptosis

Caspase-3/7 homogeneous assay was performed as described previously [3] using a kit from Promega (Madison, WI, USA). Cells were treated with garcinol or vehicle control for 72 h. After treatment, 100 μL Apo-ONE^®^ caspase-3/7 reagent was added and the plates shaken for 2 min, followed by incubation at room temperature for 3 h. The fluorescence was then evaluated using ULTRA Multifunctional Microplate Reader (TECAN) at excitation/emission wavelengths of 485/530 nm.

### 4.6. miRNA Transfections

Cells were seeded at a density of 2.5 × 10^5^ cells per well in six-well plates and transfected with appropriate anti-miRNAs or miRNA-negative controls at a final concentration of 200 nM for each individual miRNA (Ambion, Foster City, CA, USA) using DharmaFECT1 transfection reagent (Dharmacon, Lafayette, CO, USA). After 2 days of transfection, cells were split and transfected three more times (for a total four rounds of transfections) before any individual assays.

### 4.7. Real-Time RT-PCR

For miRNA analysis, total RNA was isolated using the mirVana miRNA isolation kit (Ambion). The levels of miRNAs were determined using miRNA-specific Taqman MGB probes from the Taqman MicroRNA Assay (Applied Biosystems, Waltham, MA, USA). The relative amounts of miRNA were normalized to internal miRNA controls RNU6B and RNU48.

### 4.8. Data Analysis

All the reported results are representative of at least three independent observations. The data are presented as the mean values ± SE. Statistical comparisons between groups were done using one-way ANOVA. Values of *p* < 0.05 were considered to be statistically significant and individual *p*-values are reported in the figures, as appropriate.

## Figures and Tables

**Table 1:** Garcinol lowers the IC_50_ of erlotinib/cisplatin in A549M cells.

Therapy	Garcinol (μM)	IC_50_ (μM)	% Decrease in IC_50_
Erlotinib	0	43.4	-
	5	29.1	32.95
	20	17.2	60.37
Cisplatin	0	36.5	-
	5	19.2	47.40
	20	9.1	75.07

## References

[1] Padhye S., Ahmad A., Oswal N., Sarkar F.H. (2009). Emerging role of garcinol, the antioxidant chalcone from garcinia indica choisy and its synthetic analogs. J. Hematol. Oncol..

[2] Ahmad A., Wang Z., Ali R., Maitah M.Y., Kong D., Banerjee S., Padhye S., Sarkar F.H. (2010). Apoptosis-inducing effect of garcinol is mediated by NF-κB signaling in breast cancer cells. J. Cell. Biochem..

[3] Ahmad A., Wang Z., Wojewoda C., Ali R., Kong D., Maitah M.Y., Banerjee S., Bao B., Padhye S., Sarkar F.H. (2011). Garcinol-induced apoptosis in prostate and pancreatic cancer cells is mediated by NF-κB signaling. Front. Biosci. (Elite Ed).

[4] Liu C., Ho P.C., Wong F.C., Sethi G., Wang L.Z., Goh B.C. (2015). Garcinol: Current status of its anti-oxidative, anti-inflammatory and anti-cancer effects. Cancer Lett..

[5] Tsai M.L., Chiou Y.S., Chiou L.Y., Ho C.T., Pan M.H. (2014). Garcinol suppresses inflammation-associated colon carcinogenesis in mice. Mol. Nutr. Food Res..

[6] Ranjbarnejad T., Saidijam M., Tafakh M.S., Pourjafar M., Talebzadeh F., Najafi R. (2017). Garcinol exhibits anti-proliferative activities by targeting microsomal prostaglandin E synthase-1 in human colon cancer cells. Hum. Exp. Toxicol..

[7] Duan Y.T., Yang X.A., Fang L.Y., Wang J.H., Liu Q. (2018). Anti-proliferative and anti-invasive effects of garcinol from garcinia indica on gallbladder carcinoma cells. Die Pharm..

[8] Kim S., Seo S.U., Min K.J., Woo S.M., Nam J.O., Kubatka P., Kim S., Park J.W., Kwon T.K. (2018). Garcinol enhances TRAIL-induced apoptotic cell death through up-regulation of DR5 and down-regulation of c-FLIP expression. Molecules.

[9] Siegel R.L., Miller K.D., Jemal A. (2018). Cancer statistics, 2018. CA Cancer J. Clin..

[10] Boloker G., Wang C., Zhang J. (2018). Updated statistics of lung and bronchus cancer in united states (2018). J. Thorac. Dis..

[11] Yu S.Y., Liao C.H., Chien M.H., Tsai T.Y., Lin J.K., Weng M.S. (2014). Induction of p21(WAF1/CIP1) by garcinol via downregulation of p38-MAPK signaling in p53-independent H1299 lung cancer. J. Agric. Food Chem..

[12] Huang W.C., Kuo K.T., Adebayo B.O., Wang C.H., Chen Y.J., Jin K., Tsai T.H., Yeh C.T. (2018). Garcinol inhibits cancer stem cell-like phenotype via suppression of the WNT/β-catenin/STAT3 axis signalling pathway in human non-small cell lung carcinomas. J. Nutr. Biochem..

[13] Wang J., Wang L., Ho C.T., Zhang K., Liu Q., Zhao H. (2017). Garcinol from garcinia indica downregulates cancer stem-like cell biomarker ALDH1A1 in nonsmall cell lung cancer A549 cells through DDIT3 activation. J. Agric. Food Chem..

[14] Ahmad A., Maitah M.Y., Ginnebaugh K.R., Li Y., Bao B., Gadgeel S.M., Sarkar F.H. (2013). Inhibition of hedgehog signaling sensitizes NSCLC cells to standard therapies through modulation of EMT-regulating miRNAs. J. Hematol. Oncol..

[15] Wang Z., Li Y., Ahmad A., Azmi A.S., Kong D., Banerjee S., Sarkar F.H. (2010). Targeting mirnas involved in cancer stem cell and EMT regulation: An emerging concept in overcoming drug resistance. Drug Resist. Updat..

[16] Otsuki Y., Saya H., Arima Y. (2018). Prospects for new lung cancer treatments that target EMT signaling. Dev. Dyn..

[17] Ahmad A., Sarkar S.H., Bitar B., Ali S., Aboukameel A., Sethi S., Li Y., Bao B., Kong D., Banerjee S. (2012). Garcinol regulates EMT and WNT signaling pathways in vitro and in vivo, leading to anticancer activity against breast cancer cells. Mol. Cancer Ther..

[18] Shi Z.M., Wang L., Shen H., Jiang C.F., Ge X., Li D.M., Wen Y.Y., Sun H.R., Pan M.H., Li W. (2017). Downregulation of miR-218 contributes to epithelial-mesenchymal transition and tumor metastasis in lung cancer by targeting SLUG/ZEB2 signaling. Oncogene.

[19] Ye Z., Yin S., Su Z., Bai M., Zhang H., Hei Z., Cai S. (2016). Downregulation of miR-101 contributes to epithelial-mesenchymal transition in cisplatin resistance of NSCLC cells by targeting ROCK2. Oncotarget.

[20] Park K.S., Raffeld M., Moon Y.W., Xi L., Bianco C., Pham T., Lee L.C., Mitsudomi T., Yatabe Y., Okamoto I. (2014). Cripto1 expression in EGFR-mutant NSCLC elicits intrinsic EGFR-inhibitor resistance. J. Clin. Investig..

[21] Tang J., Salama R., Gadgeel S.M., Sarkar F.H., Ahmad A. (2013). Erlotinib resistance in lung cancer: Current progress and future perspectives. Front. Pharmacol..

[22] Sarin N., Engel F., Kalayda G.V., Mannewitz M., Cinatl J., Rothweiler F., Michaelis M., Saafan H., Ritter C.A., Jaehde U. (2017). Cisplatin resistance in non-small cell lung cancer cells is associated with an abrogation of cisplatin-induced G2/M cell cycle arrest. PLoS ONE.

[23] Parasramka M.A., Ali S., Banerjee S., Deryavoush T., Sarkar F.H., Gupta S. (2013). Garcinol sensitizes human pancreatic adenocarcinoma cells to gemcitabine in association with microRNA signatures. Mol. Nutr. Food Res..

[24] Tu S.H., Chiou Y.S., Kalyanam N., Ho C.T., Chen L.C., Pan M.H. (2017). Garcinol sensitizes breast cancer cells to TAXOL through the suppression of caspase-3/IPLA2 and NF-κB/TWIST1 signaling pathways in a mouse 4T1 breast tumor model. Food Funct..

[25] Xia Y., Shen S., Verma I.M. (2014). NF-κB, an active player in human cancers. Cancer Immunol. Res..

[26] Singh A., Settleman J. (2010). EMT, cancer stem cells and drug resistance: An emerging axis of evil in the war on cancer. Oncogene.

[27] Ahmad A., Sarkar S.H., Aboukameel A., Ali S., Biersack B., Seibt S., Li Y., Bao B., Kong D., Banerjee S. (2012). Anticancer action of garcinol in vitro and in vivo is in part mediated through inhibition of STAT-3 signaling. Carcinogenesis.

[28] Li F., Shanmugam M.K., Chen L., Chatterjee S., Basha J., Kumar A.P., Kundu T.K., Sethi G. (2013). Garcinol, a polyisoprenylated benzophenone modulates multiple proinflammatory signaling cascades leading to the suppression of growth and survival of head and neck carcinoma. Cancer Prev. Res..

[29] Sethi G., Chatterjee S., Rajendran P., Li F., Shanmugam M.K., Wong K.F., Kumar A.P., Senapati P., Behera A.K., Hui K.M. (2014). Inhibition of STAT3 dimerization and acetylation by garcinol suppresses the growth of human hepatocellular carcinoma in vitro and in vivo. Mol. Cancer.

[30] Ahmad A., Li Y., Sarkar F.H. (2016). The bounty of nature for changing the cancer landscape. Mol. Nutr. Food Res..

[31] Padhye S., Ahmad A., Oswal N., Dandawate P., Rub R.A., Deshpande J., Swamy K.V., Sarkar F.H. (2010). Fluorinated 2′-hydroxychalcones as garcinol analogs with enhanced antioxidant and anticancer activities. Bioorg. Med. Chem. Lett..

[32] Gaonkar R.H., Ganguly S., Dewanjee S., Sinha S., Gupta A., Ganguly S., Chattopadhyay D., Chatterjee Debnath M. (2017). Garcinol loaded vitamin E TPGS emulsified PLGA nanoparticles: Preparation, physicochemical characterization, in vitro and in vivo studies. Sci. Rep..

[33] Li F., Shanmugam M.K., Siveen K.S., Wang F., Ong T.H., Loo S.Y., Swamy M.M., Mandal S., Kumar A.P., Goh B.C. (2015). Garcinol sensitizes human head and neck carcinoma to cisplatin in a xenograft mouse model despite downregulation of proliferative biomarkers. Oncotarget.

[34] Saadat N., Akhtar S., Goja A., Razalli N.H., Geamanu A., David D., Shen Y., Gupta S.V. (2018). Dietary garcinol arrests pancreatic cancer in p53 and K-ras conditional mutant mouse model. Nutr. Cancer.

[35] Mantelingu K., Reddy B.A., Swaminathan V., Kishore A.H., Siddappa N.B., Kumar G.V., Nagashankar G., Natesh N., Roy S., Sadhale P.P. (2007). Specific inhibition of p300-HAT alters global gene expression and represses HIV replication. Chem. Biol..

